# Wavelet-Based Watermarking and Compression for ECG Signals with Verification Evaluation

**DOI:** 10.3390/s140203721

**Published:** 2014-02-21

**Authors:** Kuo-Kun Tseng, Xialong He, Woon-Man Kung, Shuo-Tsung Chen, Minghong Liao, Huang-Nan Huang

**Affiliations:** 1 Department of Computer Science and Technology, Harbin Institute of Technology Shenzhen Graduate School, Shenzhen 518055, China; E-Mails: kuokun.tseng@gmail.com (K.-K.T.); xialong@gmail.com (X.H.); 2 Department of Exercise and Health Promotion, College of Education, Chinese Culture University (CCU) and Department of Neurosurgery, Lo-Hsu Foundation, Lotung Poh-Ai Hospital, Luodong, Yilan 265, Taiwan; E-Mail: nskungwm@yahoo.com.tw; 3 Department of Applied Mathematics, Tunghai University, Taichung 40704, Taiwan; 4 Department of Software Engineering, Xiamen University, Xiamen 361005, China; E-Mail: minghong@gmail.com; 5 Department of Applied Mathematics, Tunghai University, Taichung 40704, Taiwan; E-Mail: nhuang@thu.edu.tw

**Keywords:** integrating, watermarking, ECG, compression

## Abstract

In the current open society and with the growth of human rights, people are more and more concerned about the privacy of their information and other important data. This study makes use of electrocardiography (ECG) data in order to protect individual information. An ECG signal can not only be used to analyze disease, but also to provide crucial biometric information for identification and authentication. In this study, we propose a new idea of integrating electrocardiogram watermarking and compression approach, which has never been researched before. ECG watermarking can ensure the confidentiality and reliability of a user's data while reducing the amount of data. In the evaluation, we apply the embedding capacity, bit error rate (BER), signal-to-noise ratio (SNR), compression ratio (CR), and compressed-signal to noise ratio (CNR) methods to assess the proposed algorithm. After comprehensive evaluation the final results show that our algorithm is robust and feasible.

## 1. Introduction

The Internet is not only brings us convenience, but also risks. The topic of individuals' privacy is attracting more and more attention. Electrocardiograms as personal data are being applied more and more as a biometric [1] and deserve to be protected. At the same time, the use of the Internet is ncreasing and its carrying capacity is being tested like never before. Therefore, in this paper we propose a method based on wavelets to add watermarks to electrocardiograms and compress them. We expect to reduce the pressure on the Internet and preserve the ECG characteristics while protecting the security of ECG data in network transmission [2].

An ECG reflects the process of the electrical activity of the heart, which can be taken as a reference for the study of cardiac function and cardiac pathology [3]. With an ECG signal, we can analyze and identify various arrhythmias, and understand the degree and development of myocardial damage, as well as the structure and function of the atrium and ventricle. Besides, it is necessary to decrease the demand for the ECG data storage capacity and data transmission bandwidth [4]. Accordingly, we integrated the quantization based digital watermarking with a new compression method, which is used to watermark the ECG signal and compress the data, while allowing the watermark to be verified. The watermark can ensure the security of the ECG signal and enable it to be restored to its original state. At the same time, we proposed a wavelet compression method to achieve lossy compression of the ECG signal. By removing the high frequency portion under different wavelet basis, we can ensure the compression rate and accuracy. The compression rate is around 1.96. After watermarking and compression, it can be aligned for transmission. On the receiving side, the data decompression and the watermark extraction can then be finished. In summary, we make the following contributions: we integrate electrocardiogram digital watermark encryption and a compression algorithm based on an orthogonal wavelet domain, which has never been researched before.

This study is organized as follows: in Section 2, we introduce background knowledge and related research. In Section 3, we introduce the architecture and the basic algorithm of the proposed method, including the digital watermark, wavelet transform and compression formulas. In Section 4, we introduce the evaluation method. This is mainly a comparison of the watermarked and compressed object before and after, as well as comparison of the correlation peaks. Some conclusions are drawn in Section 5.

## 2. Background and Related Work

### 2.1. ECG Algorithm Review

There are currently no ECG studies which include research on both watermarks and compression. However, there are some studies looking at compression or watermarking individually, so based on existing research, we surveyed watermarking and compression as two separate aspects.

**Figure 1.**
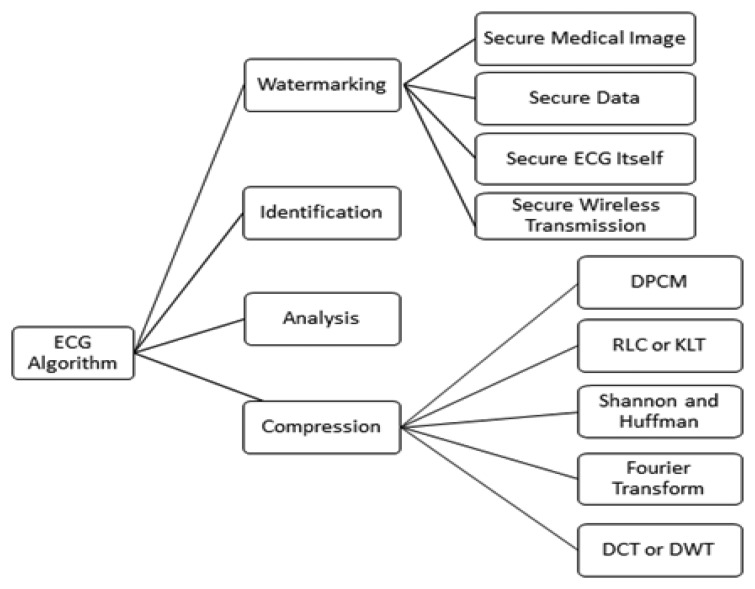
Related works.

At present, from the watermark point of view, research on the protection of ECG information is still in its infancy, although there are some research studies, shown in Figure 1, related to the watermarking of ECG signals, and with the use of wavelet transform based digital watermarking encryption technology [5]. Therefore, research in this field has great potential for the researcher. The existing research may be divided several categories.

The first application is the digital watermark technology used in medical images. This application proposes a novel blind watermarking method, by embedding a secret key into the medical image of ECG signals. The second is a sensor network-based ECG monitoring system. ECG signals are watermarked with patient biomedical information to confirm patient/ECG linkage integrity [6]. The third application is wavelet transform-based ECG digital watermarking technology. In ECG signals, the energy is concentrated in QRS complex waves [7], so the selection of wavelet coefficients for concealment should avoid causing the QRS complex waves to distort obviously. The last application is ECG transmission in a wireless network. This paper proposes the use of digital watermarking to ensure the safe transmission of ECG signals in a wireless network [8]. A low frequency chirp signal is used to embed the watermark, which is a 15-bit digital code assigned to the patient. The characteristic of the proposed watermarking scheme is that the embedded watermark can be fully removed by the receiver due to the blind recovery feature of the watermark [9]. Dey *et al.* [10] proposed a novel session based blind watermarking method with a secret key by embedding a binary watermark image into the ECG signal. In addition, the “P Q R S T”-peaks are marked and stored over the entire ECG signal and the time interval between two consecutive ‘R’-peaks, and intervals between other peaks, are measured to detect anomalies in the behavior of the heart. However, these two methods are non-blind. Ayman and Ibrahim proposed a wavelet-based steganography technique which combines encryption and scrambling technique to protect patient confidential data. The proposed method allows the ECG signal to hide its corresponding patient confidential data and other physiological information [11].

In the aspect of ECG compression, the ECG is a dynamic signal. It will continue to produce new signals. For example, Holter monitoring technology has been applied more and more, and there are patients for whom more than 24 h of ECG data has to be collected, which greatly increases the amount of data you need to record. With the advent of an aging society, the number of patients with heart disease will grow, and cardiac care will become a social problem. Remote transmission of ECGs can allow real-time monitoring; it is conducive to diagnosis and first aid instructions. Therefore, the remote transmission of ECGs has a good economic and market outlook [12]. ECG signal compression is a key technology for remote ECG transmission. It directly determines the practicality and effectiveness of the system.

For example, in a wireless communication network which is employed for data transmission, long term ECG guardianship generates a huge amount of data that will make wireless communication costs unacceptable, and raise issues of transmission speed and bandwidth. ECG signal compression technology will guarantee that none of the information of the ECG signal is lost and will minimize the amount of data that needs to be transmitted, reduce transmission costs, and increase transmission speed.

With the intervention of computer technology, ECG data compression technology is increasingly showing its importance. The Holter data compression algorithm is one of the most fruitful hotspots of current international research in the field of biomedical signal processing [13]. Data compression is possible with a variety of methods. Early predictive coding methods, such as Differential Pulse Code Modulation (DPCM), directly encode the amplitude variation of the adjacent sample values. The principle of these methods is simple and easy to implement, but the compression rate is relatively low. Run-length coding (RLC) uses the correlation among the symbols, by recording the length of each symbol to achieve compression. Shannon-Fano codes and Huffman codes are based on the frequency with which each signal appears [14]. Then they assign the most economical code length so as to achieve compression. With a flat distribution of the signal in the time domain, after orthogonal transformation, the energy will be concentrated on the low-frequency component so the high-frequency component can be omitted, or we can use only a few bits to encode them. These transform compression methods include the Karhunen-Loeve transform (KLT), Fourier Transform (DFT, FFT), and discrete cosine transform (DCT) [15]. New compression techniques include the neural network and wavelet transform (DWT) methods and others [16]. Two algorithms are described that are suited for real-time biomedical signal compression, these being amplitude threshold compression and SQ segment compression [17].

### 2.2. Discrete Wavelet Transform

Wavelets are obtained by a single prototype function (mother wavelet) *ψ(x)* which is regulated with a scaling parameter and a shift parameter. It maps the function in *L^2^(R)* onto a scale-space plane. The discrete normalized scaling and wavelet basis function are defined as

(1) $$\varphi_{i,n}(t)=2^{\overset{i}{\;}/\underset{2}{\;}}h_{i}\varphi (2^{i}t-n)$$

(2) $$\psi_{i,n}=2^{\overset{i}{\;}/\underset{2}{\;}}g_{i}\psi (2^{i}t-n)$$

where *i* and n are the dilation and translation parameters; *h_i_* and *g_i_* are the low-pass and high-pass filters. Orthogonal wavelet basis functions not only provide simple calculation in coefficients expansion but also span *L^2^(R)* in signal processing. As a result, any digital signal *S(t)*∈*L^2^(R)* can be expressed as a series expansion of orthogonal scaling functions and wavelets. More specifically:

(3) $$S(t)=\sum_{t}c_{j_{0}}\;(t)\varphi_{j_{0},t}(t)+\sum_{k}\sum_{j=j_{0}}^{\infty }d_{j}\;(k)\psi_{j,k}(t)$$

where:

(4) $$c_{j}\;(t)=\int S(t)\varphi_{j,l}(t)dt$$

(5) $$d_{j}\;(k)=\int S(t)\psi_{j,k}(t)dt$$

**Figure 2.**
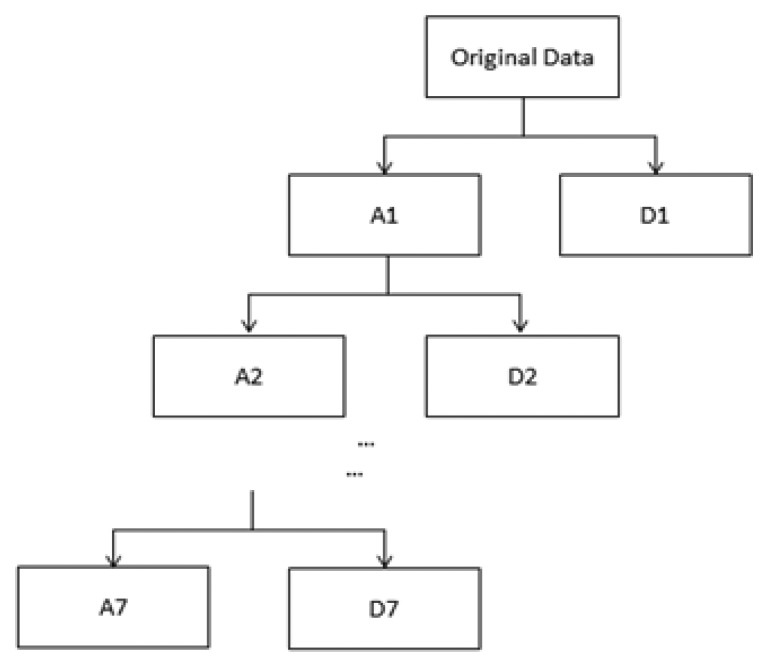
Decomposition level.

They denote the sequences of low-pass and high-pass coefficients, respectively; *j_0_* is the integer which defines an interval on which *S(t)* is piecewise constant [18]. Throughout this paper, the host digital ECG signal *S(n), n*∈*R*, denoting samples of the original ECG signal *S(t)* at the nth sample time, is cut into segments where DWT will be performed. This can be done by exploiting the Haar wavelet with an orthogonal basis to implement DWT through a filter bank. Figure 2 demonstrates how the input digital ECG signal *S(n)* is decomposed into eight non-overlapping multi-resolution sub-bands by the seven-level DWT decomposition.

## 3. Proposed Architecture and Algorithm

This section introduces the proposed architecture and algorithm. The first is data preparation of the ECG signal. Digital watermark insertion and extraction are discussed in Section 3.2. The proposed data compression is introduced in Section 3.3.

### 3.1. Data Preparation

ECG refers to the heart in each cardiac cycle, in which tracings of the pacemaker, atrial and ventricular function successively excited one by one, along with the bioelectrical changes monitored in the ECG, lead to the graphics of the various forms of potential changes detected from the surface (referred to as ECG) [19]. The ECG provides objective indicators of when the heart is excited about an occurrence, its spread and the recovery process. The ECG shows the electrical activity of the excited heart, and it has an important reference value in basic functions of the heart and pathology research. The ECG can be used to analyze and identify a variety of arrhythmias; it can also reflect the extent and development of myocardial damage and atrial and ventricular function and structural condition [20]. It has reference value in guiding cardiac surgery and suggests the necessary drug treatment. The standard ECG leads to electrocardiogram waves, named by the Dutch physiologist W. Einthoven, the inventor of the ECG [21]. He divided one cardiac cycle into P, Q, R, S, and T waves.

There are currently three internationally recognized ECG databases which can be used as a standard, namely, the Massachusetts Institute of Technology's MIT-BIH Arrhythmia Database, the AHA database of the American Heart Association (AHA) and the European ST-T ECG database [22]. In this paper, we selected the ECG data from the MIT-BIH Arrhythmia database. This database includes 48 groups, with two-lead ECG recordings for half an hour, a total of up to 24 h of information. This database contains 47 individuals' ECG information (datasets ID 201 and 202 are duplicated, so we select different signal segments for our test); the subjects consist of 25 men aged between 32 to 89 and 22 women aged from 23 to 89. These ECG data have a sampling rate of 360 Hz and a 12-bit binary representation. Each ECG signal is first adjusted to have zero mean to eliminate any DC offset.

### 3.2. Digital Watermark Insertion and Extraction

Digital watermarking technology refers to directly embedding some identifying information (digital watermark) into the digital carrier (including multimedia, documents, software, *etc.*) so that it does not affect the usage value of the original carrier and is hard to be perceived or noticed with by people's perception systems (such as visual or auditory systems) [23]. The information hidden in the carrier can help us confirm the content creators, buyers, carriers transmitting secret information, and determine whether the carrier has been altered. Digital watermarking is an important research direction in information-hiding technology.

**Figure 3.**
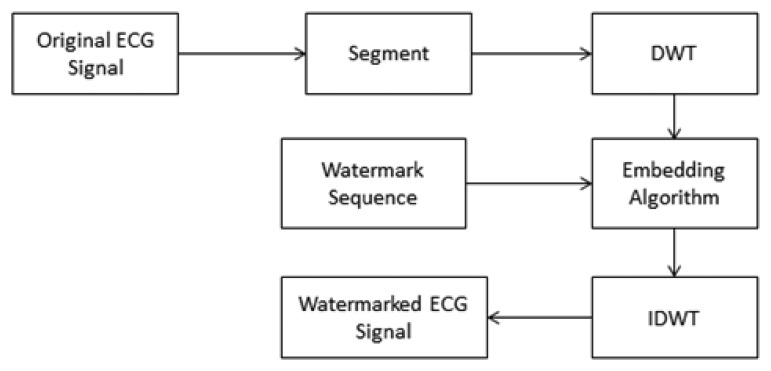
Watermark embedding model.

ECGs have high accuracy requirements for heart disease diagnosis. It is necessary to maintain the shape of the ECG waveforms in watermarking since the ECG diagnosis mainly depends on the PQRST waves. To achieve this goal, we use quantization-based digital watermark encryption technology on the electrocardiogram (ECG) to protect patient rights and information. First of all, the ECG signal is cut into several segments. The segment length depends on the level of wavelet decomposition. As shown in Figure 2, we then perform seven levels of wavelet decomposition on each segment so that the input ECG signal is decomposed into eight non-overlapping sub-bands. Taking into account the robust performance of the low-pass filtering, we embedded the watermark sequence with patient's information into the lowest frequency wavelet coefficients in level seven. The watermark sequence with patient's information {*m_i_*} is embedded by the following rule:

(6) $$[{c^{\prime }}_{i}=\{\begin{array}{l} \left\lfloor \overset{c_{i}}{\;}/\underset{T}{\;}\right\rfloor T+\overset{3T}{\;}/\underset{4}{\;},\text{if}\;m_{i}=1\\ \left\lfloor \overset{c_{i}}{\;}/\underset{T}{\;}\right\rfloor T+\overset{T}{\;}/\underset{4}{\;},\text{if}\;m_{i}=0 \end{array}]$$

where *{c_i_}* and *{c_i_′}* are the original and the watermarked DWT coefficients; *T* is the embedding strength. After embedding the watermark sequence with patient's information into the DWT lowest-frequency sub-band of the processed ECG signal, then the watermarked ECG signal is obtained through inverse DWT. Figure 3 shows the embedding model.

In order to adjust a proper embedding strength *T*, we consider the signal to noise ratio (SNR) which is defined by [24,25]:

(7) $$\left[SNR=-10\log_{10}\;\left\{\frac{\sum_{n}{\left(\tilde{S}(n)-S(n)\right)}^{2}}{\sum_{n}{\left(S(n)\right)}^{2}}\right\}\right]$$

where *S(n)* and $\tilde{S}(n)$ are the original and the watermarked audio.

Due to the fact that the DWT coefficients are implemented with orthogonal wavelet bases and according to Parseval's theorem, the energy in a signal is given as follows:

(8) $$[\int {\left|S(t)\right|}^{2}dt=\sum_{l=-\infty }^{\infty }{\left|c(l)\right|}^{2}+\sum_{j=0}^{\infty }\sum_{k=-\infty }^{\infty }{\left|d_{j}\;(k)\right|}^{2}]$$

Since the high frequency sub-band in Equation (8) are neglected by the proposed embedding algorithm, only the lowest DWT coefficients are adjusted. That is

(9) $$\left[\text{SNR}=-10\log\left(\frac{\sum {\left\|c_{i}-{c^{\prime }}_{i}\right\|}_{2}^{2}}{\sum {\left\|c_{i}\right\|}_{2}^{2}+\sum {\left(\text{high frequency sub}-\text{band DWT coefficients}\right)}^{2}}\right)\approx -10\log(\frac{\sum {\left\|c_{i}-{c^{\prime }}_{i}\right\|}_{2}^{2}}{\sum {\left\|c_{i}\right\|}_{2}^{2}})\right]$$

We use this formula to adjust the embedding strength *T.*

When extracting the hidden data, we first divide the watermarked ECG signal into the same segments in the embedding manner. Then, we perform DWT on each segment which has been embedded with a watermark. The watermark is extracted from the DWT lowest-frequency sub-band as follows. Suppose $\left\{c_{i}^{*}\right\}$ is the coefficient of the lowest-frequency sub-band; we use the following rule to extract watermark sequence $\left\{m_{i}^{*}\right\}$ From $\left\{c_{i}^{*}\right\}$ :

(10) $$[m_{i}^{*}=\{\begin{array}{c} 1,\text{if}\;c_{i}^{*}-\left\lfloor \overset{c_{i}^{*}}{\;}/\underset{T}{\;}\right\rfloor T\geq \;\overset{T}{\;}/\underset{2}{\;}\\ 0,\text{if}\;c_{i}^{*}-\left\lfloor \overset{c_{i}^{*}}{\;}/\underset{T}{\;}\right\rfloor T<\;\overset{T}{\;}/\underset{2}{\;} \end{array}]$$

**Figure 4.**
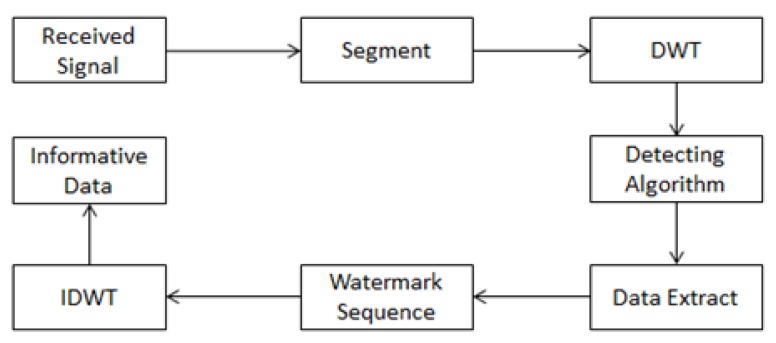
Watermark extraction model.

After we determine the location of the watermark sequence, we can extract the hidden information. The extraction model is shown in Figure 4.

### 3.3. Wavelet Transform of Data Compression

**Figure 5.**
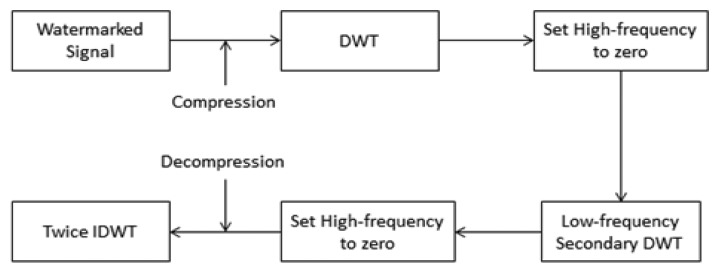
Data compression model.

Signal processing has become an important topic in contemporary science and technology. The aims of signal processing are accurate analysis, diagnosis, compression coding and quantization, storage, and signal recovery [26]. Currently, the ideal tool for stationary signal analysis is still the Fourier transform [27]. However, in practical applications, the vast majority of signals are non-stationary, so Fourier analysis is not suitable. The ECG signal mentioned in this study is a typical non-stationary signal, and wavelet theory of multi-resolution analysis for ECG signal processing is a new idea. Compared with other time-frequency analysis, the advantages of wavelet theory are that it cannot only adapt to the time-frequency resolution characteristics of non-stationary signals, but also decompose signals on an orthogonal basis. It is also easy to describe the non-stationary signals' time-frequency characteristics with a small number of parameters. These all constitute excellent features for an extraction algorithm. Figure 5 is a compression flow diagram of the ECG signal.

In the first compression, we use the wavelet function bior1.1. In the second compression, we use the wavelet function bior3.7. Therein, bior can also be expressed as bior Nr. Nd. Nr and Nd are related parameters for remodeling and decomposition filter length. The bior wavelet is a biorthogonal wavelet. *c_1_ (n) (n∈Z)* is the input of the filter, i.e., a watermark signal. After the conversion, the middle output is:

(11) $$[c_{0}\;(k)=\sum_{n}\tilde{h}(2k-n)c_{1}\;(n)]$$

(12) $$[d_{0}\;(k)=\sum_{n}\tilde{g}(2k-n)c_{1}\;(n)]$$

Based on the output of the filter group:

(13) $$[{\tilde{c}}_{1}\;(m)=\sum_{k}\left[h(2k-m)c_{0}\;(k)+g(2k-m)d_{0}\;(k)]\right]$$

With merger and exchange, we can get the following formula:

(14) $$[{\tilde{c}}_{1}\;(m)=\sum_{n}\sum_{k}\left[h(2k-m)\tilde{h}(2k-n)+g(2k-m)\tilde{g}(2k-n)]c_{1}\;(n)\right](14)$$

In order to fully reconstruct, even with the expression:

(15) $$\left[{\tilde{c}}_{1}\;(m)=c_{1}\;(m)(\forall m\in Z)\right]$$

we need the following equation to be established:

(16) $$[\sum_{k}\left[h(2k-m)\tilde{h}(2k-n)+g(2k-m)\tilde{g}(2k-n)]=\delta (m-n)\right]$$

The wavelet method for compression plays a role in optimizing the waveform of the electrocardiogram. We choose to remove the high frequency components. The change in the generated waveform has no effect on the doctor's diagnosis. Instead, it is possible to make the waveform more easily identifiable. In this way, we reduce not only the transmission volume, but also the noise of the ECG.

## 4. Evaluation

In this section, we execute watermark encryption and compression on each ECG signal with length 4,096 sampled from 47 datasets in the MIT-BIH arrhythmia database. Each ECG signal is first adjusted to have zero mean to eliminate the DC offset and then the Haar wavelet transform is applied to each signal with 7-level decomposition. Evaluation of the watermarked and compressed ECG signals is presented in the following sections.

### 4.1. Embedding Capacity

The embedding capacity refers to the number of bits which are embedded in the ECG signal. Since we embedded the watermark sequence with patient information into the lowest frequency wavelet coefficients in level seven, the embedding capacity is calculated as 4096÷2^7^=32(bits).

### 4.2. Robustness Testing under Fixed SNR

**Figure 6.**
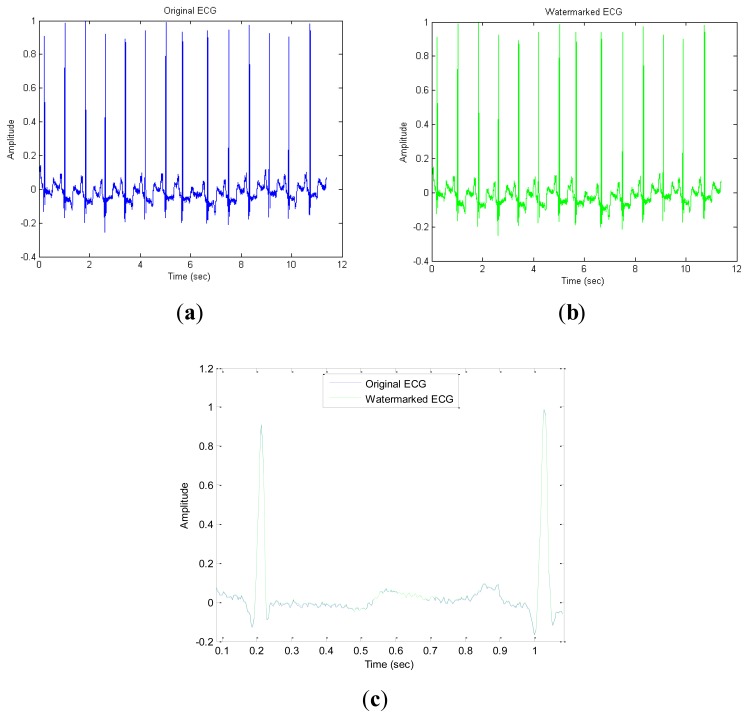
The original and watermarked ECG. (**a**) Watermarked signal (**b**) Original signal; (**c**) Waveform comparison between 0.09 and 1.09 (s).

After the embedding process, some common attacks are applied to test the robustness, which will be measured by the bit error rate (BER). The BER, that is the ratio of bit errors to the total transferred errors during a tested time interval, is usually expressed as a percentage and can be formulated as:

(17) $$\text{BER}=\frac{B_{\text{error}}}{B_{\text{total}}}\times 100\%$$

where *B_error_* and *B_total_* denote the numbers of error binary bits and total binary bits during a tested period. In general, the performance of a watermarking system is analyzed in terms of SNR and BER. However, there is a tradeoff between them. Accordingly, we compare our results with reference [10] which used a spread-spectrum technique under the same SNR = 32 dB conditions. In order to maintain the consistency of the watermarked signal and the original signal to the maximum extent possible since the insertion of the watermark will affect the original ECG signal, we use the formula in Equation (9) to obtain the proper embedding strength *T* = 4,000. Figure 6a shows that the original and watermarked signals for data set ID 100 look almost indistinguishable. Here the blue curve represents the original ECG signals; and the green curve represents the watermarked ECG signals. We enlarge the portion in Figure 6a and b around the first second and plot both on the same graph as drawn in Figure 6c which indicates that the difference before and after watermarked signals is almost negligible in the time domain.

The results of testing three attacks are discussed in the following section:

(1) *Noise corruption*: Since the ECG data may be transferred using a network, we first consider the white noise attack to test the robustness, i.e., ${\text{S}}_{i}^{*}=\tilde{S_{i}}+\alpha \cdot y_{i}$ where $\left\{{\tilde{\text{S}}}_{i}\right\}$ is the watermarked signal and $\left\{S_{i}^{*}\right\}$ is the attacked signal which is influenced by the white noise {*y_i_*} with zero mean and standard deviation one. Here *α* is considered as the strength of the white noise, i.e.,{*αy_i_*} is zero-mean white noise with standard deviation *α*. Table 1 describes the robustness of the watermarked ECG signal under white noise attack with different standard deviations.
(2) *Low-pass filtering*: Table 2 shows the effect of using a low-pass filter with a cutoff frequency of 90, 100 or 140 Hz. The proposed method has lower robustness against the low-pass filter attack than the reference [10] at 90 and 100 Hz.
(3) *Re-sampling*: The sampling rate of the watermarked ECG signals was down-sampled from 360 Hz to 180 Hz and then back to 360 Hz using interpolation. Besides, we also adjust the sampling rate from 360 Hz to 90 Hz, 45 Hz, respectively, and then back to 360 Hz. Table 3 shows the results of these re-sampling processes, which indicate that the proposed scheme is more robust than that described in reference [10].

### 4.3. The Quality Evaluation of Compression

**Figure 7.**
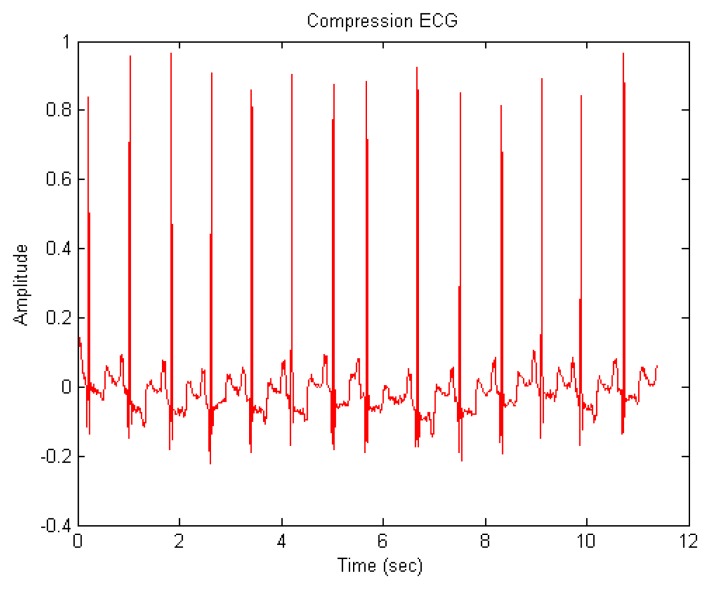
The ECG signal after compression.

We perform the proposed ECG signal compression using the MATLAB numerical simulation software. Figure 7 shows the ECG signal after compression. Some indices including compression ratio (CR), compressed-signal to noise ratio (CNR), and are utilized to evaluate the quality of compression.

The compression ratio (CR) is defined as the size (data storage bytes) ratio of the original signal to that of the compressed signal [28]:

(18) $$\left[\text{CR}=\frac{\text{Data size before compression}}{\text{Data size after compression}}\right]$$

**Figure 8.**
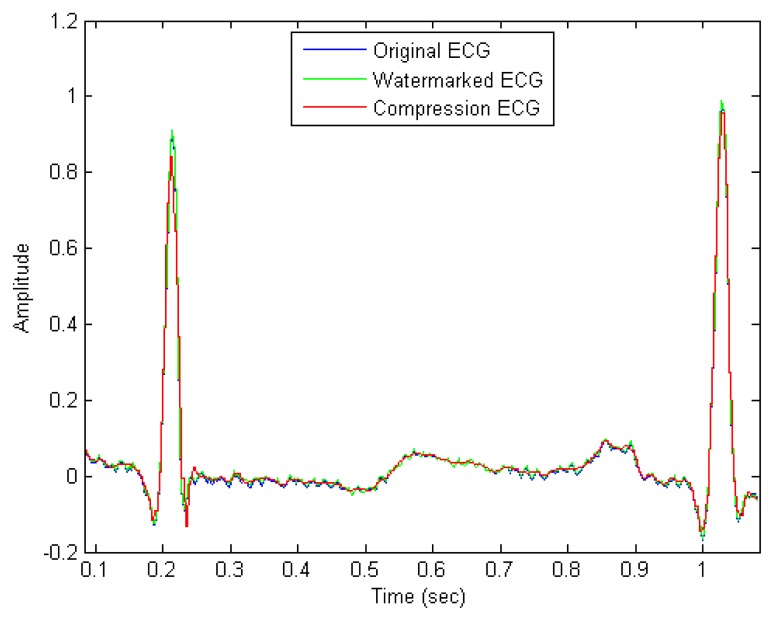
The original, watermarked, and compression ECG signal; the blue curve indicates the original, the green curve represents the watermarked, and the red curve represents the compressed.

The CR of the first compression (CR1) and the CR of the second compression (CR2) are listed in the Table 4. Compressed-signal to noise ratio (CNR) is proposed to evaluate the quality of compressed ECG signal and is defined as follows:

(19) $$\left[CNR=10\log[\frac{\sum_{i=1}^{n}{s_{i}}^{2}}{\sum_{i=1}^{n}{\left(s_{i}-D_{i}\right)}^{2}}]\right]$$

where *S* represents the source data and *D* represents the data after compression. The CNR of the first compression (CNR1) and the CNR of the second compression (CNR2) are listed in Table 4. Figure 8 shows the original, watermarked, and compression ECG signal. The blue curve indicates the original, the green curve represents the watermarked, and the red curve represents the compressed. As shown in the figure, the differences among the original signal, the watermarked signal and the decompression signal are so small as to be almost negligible.

In addition to the previous evaluation methods, verification evaluation is proposed to measure the similarity between the original ECG signal *S*_1_ and the watermarked (or compressed) ECG signal *S*_2_. First, we obtain watermarked and compression ECG data and then perform ECG verification for the 47 individual data sets for a fair comparison. The evaluation process can be divided into the following three steps:

First, we must preprocess the evaluation data from the previous watermarking and compression approaches. It first segments the previous watermarking and compression ECG signals, and processes them into a binary signal for length consistency. This uses a sampling rate of 360 with four cycles as a group, and every four groups form a sequence. If the cycle is insufficient, it makes a copy of the first cycle for the insufficient cycle. Moreover, if the ECG signal of each cycle is not consistent, we also perform a stretch or shrink for length consistency.

In the second step, we perform the DWT on the previously preprocessing data, respectively. In each level of the wavelet transform, we take its lowest-frequency part, and do the next level wavelet transform with it, until eight layers of transformation are completed. At this point, we have the data that we will deal with. In the final step, we want to compare the similarity of two processed ECG data sets. Assuming two ECG signal segments *E*_1_ ∈ *S*_1_ and *E*_2_ ∈ *S*_2_ are in different sections, we designed a method to determine their similarity. Here is our weighted distance formula used to determine the similarity of signals *E_1_* and *E_2_*:

(20) $$[d(E_{1},E_{2})=\frac{\sum_{i=0}^{\left(2^{n}-1\right)}\left|R_{1}(i)-R_{2}(i)\right|s_{1}(i)s_{2}(i)}{(2^{n}-1)\sum_{i=0}^{\left(2^{n}-1\right)}s_{1}(i)s_{2}(i)}$$

where *E* represents an ECG segment of one group, which is the basic unit for data analysis; *s* represents the relative coefficients from DWT respectively; *R* represents the rank of *i* in the sequence *E; i* is 1 or 2.

**Figure 9.**
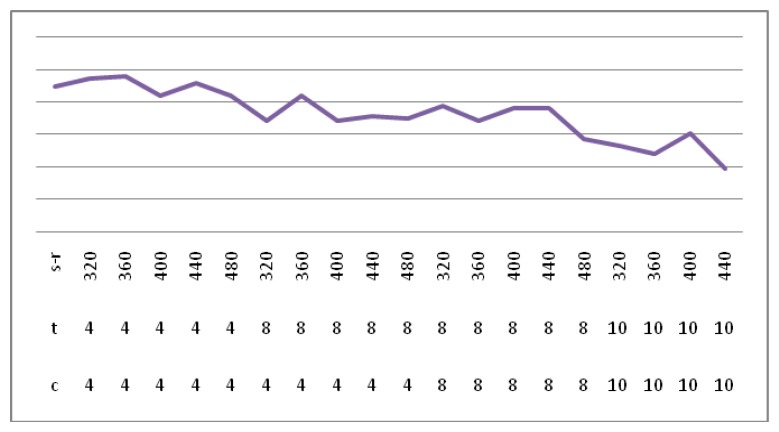
The ECG verification success rate.

Figure 9 shows the verification success rate in a different sample rate, team and cycle. The final selection of grouping is 4, the period is 4, and the sampling rate is 400. In Figure 9, the highest success rate obtained is 0.9901, where s-t is the sampling rate, c represents the cycle, t indicates the team.

## 5. Conclusions

In this study, we have designed and implemented a wavelet analysis of an ECG watermark and compression algorithm. This technology can be used to protect ECG transmission security, and reduce the transmission volume, while optimizing the ECG shape. In order to guarantee the security of the watermark embedded in the signal wavelet decomposition, the watermark was embedded in the lowest-frequency coefficients. For a uniform robustness test, we ran 48 datasets from the same database. In comparison with other methods, our method not only provides a better SNR after embedding the watermark, but also has stronger robustness than the other. For compression, we chose another wavelet function, and then we removed the high-frequency part of the wavelet decomposition. Its impact on the ECG shape is small. In addition to the integrated watermarking and compression idea, our proposed approach has the obvious advantages of robustness and ECG verification evaluation.

## Figures and Tables

**Table 1.** Testing Results via White Noise Attack with various strength.

**Strength *α***	**The Proposed Algorithm**	**Reference [10]**
150	0	0
200	0	3.125
250	0	6.25

**Table 2.** Testing results via low-pass filtering attack with various cut-off frequencies.

**Cut-off Frequency (Hz)**	**The Proposed Algorithm**	**Reference [10]**
140	0	6.25
100	11.76	9.375
90	11.76	9.375

**Table 3.** Testing results via resampling attack with various sampling rates.

**Sampling Rate**	**The Proposed Algorithm**	**Reference [10]**
1/2	0	0
1/4	0	0
1/8	0	3.125

**Table 4.** Testing Results of CR1, CR2, CNR1, and CNR2.

**CR1**	**CR2**	**CNR1**	**CNR2**
1.28	1.96	16.2563	14.7179
